# Complications of image-guided liver biopsies: Results of a nationwide database analysis

**DOI:** 10.1371/journal.pone.0323695

**Published:** 2025-06-02

**Authors:** Markus Graf, Christiana Graf, Sebastian Ziegelmayer, Alexander W. Marka, Marcus Makowski, Yannick Teumer, Philipp Paprottka, Nele Willemsen, Jonathan Nadjiri

**Affiliations:** 1 Department of Diagnostic Radiology, School of Medicine and Klinikum rechts der Isar, Technical University of Munich, Munich, Germany; 2 Department of Internal Medicine II, LMU University Hospital Munich, Munich, Germany; 3 Department of Cardiology, Ulm University Heart Center, Ulm, Germany; 4 Department of Interventional Radiology, School of Medicine and Klinikum rechts der Isar, Technical University of Munich, Munich, Germany; Institute for Clinical and Experimental Medicine, CZECHIA

## Abstract

**Background and aims:**

Liver biopsy is the gold standard for evaluating liver diseases, the diagnosis of liver fibrosis or liver cirrhosis and malignancy. However, it is susceptible to complications, and safety data on liver biopsies remain scarce. The following study examined the complication rates following percutaneous liver biopsies.

**Methods:**

We performed a study using data collected by the German interventional radiology society (DeGIR) from 2018 to 2021 of elective percutaneous liver biopsies. Clinical and hematological parameters, technical features and adverse events were retrospectively examined.

**Results:**

From 2018 to 2021, a total of 12117 percutaneous liver biopsies were performed in 194 participating centers in Germany. Complications occurred in 235 biopsies (1.9%), of which 195 (1.6%) were major adverse events. Minor complications in the form of procedural hypotension and pain occurred in 7 and 33 cases (0.06% and 0.3%, respectively). Major complications such as bleeding, organ injury and pneumothorax were observed in 166, 3 and 26 cases (1.4%, 0.02% and 0.2%). Three subjects (0.02%) died as a result of massive intraperitoneal bleeding. Major and bleeding complications were significantly more frequently observed in patients with thrombocytopenia (p < 0.001) as well as in patients undergoing computed tomography (CT)-guided procedure compared to ultrasound-guided one (p < 0.001). Moreover, general and bleeding complication rates significantly differed by the liver segment biopsied (p < 0.001). In contrast, the type of needle size used (p = 0.323), internationalized ratio (INR) (p = 0.09), aPTT (p = 0.98), gender (p = 0.83), age (p = 0.08) and the number of biopsies (p = 0.91) performed did not impact the frequency of major adverse events. By multivariate logistic regression analysis, platelet count, the imaging modality used (CT vs. ultrasound-guided) and the liver segment biopsied were identified as independent risk factors of post-biopsy bleeding (p < 0.001 each).

**Conclusion:**

Percutaneous liver biopsies are safe with rare procedural morbidity. Our data confirm previous data by showing that post-procedural bleeding was not associated with INR and aPTT in patients undergoing invasive procedures. However, measurement of platelet count is indicated to identify patients with increased procedural bleeding risk. Moreover, our findings suggest that patients with liver cirrhosis as well as patients with complex findings and difficult localizations could benefit from intensified monitoring post-procedural.

## Introduction

Despite significant advances in non-invasive diagnostic techniques for liver assessment, including ultrasound, computed tomography (CT), magnetic resonance imaging (MRI), and techniques for measuring liver stiffness such as vibration-controlled transient elastography (TE) and MR elastography, liver biopsy still retains its essential role [1–3]. One of the reasons is that imaging techniques, while advanced, can sometimes produce inconclusive results or be limited by patient factors such as obesity or the presence of ascites [4,5]. In addition, the overlapping nature of imaging findings in many liver diseases makes it difficult to make a definitive diagnosis based on imaging results alone. Although these advanced imaging modalities are extremely valuable in their sensitivity for detecting structural liver changes and assessing liver stiffness, they lack the histopathologic specificity required for definitive diagnosis, particularly in the case of liver tumors [6–8]. In this context, liver biopsy, complemented by detailed histological examination, remains essential for definitive histopathological confirmation of intrinsic liver malignancies such as hepatocellular carcinoma (HCC) and various metastatic diseases. This step is fundamental as it defines the diagnostic pathway, determines therapeutic strategies and has a direct impact on patient prognosis [9].

The importance of liver biopsy in the management of HCC is well established, and it highlights new advances and difficulties in this area. Despite the establishment of various treatment modalities, liver biopsy remains the cornerstone of HCC management, providing key data to guide treatment decisions [10].

Moreover, liver biopsy remains a mainstay in the investigation of unexplained liver pathology, establishing the diagnosis of hepatic manifestations of systemic diseases such as sarcoidosis or graft-versus-host disease, and is an integral part of the management of diseases such as autoimmune hepatitis or variant syndromes of autoimmune disease [11,12]. In the setting of acute to chronic liver failure (ACLF) due to autoimmune liver diseases, liver biopsy may be helpful in confirming the diagnosis, particularly as a large proportion of cases may be seronegative. Histopathology is often required to confirm the diagnosis and to initiate appropriate corticosteroid therapy, which has been shown to improve survival in these patients [13].

According to the recently published clinical practice guidelines of the European Association for the Study of the Liver (EASL), percutaneous as well as transjugular biopsy are classified as procedures associated with a low risk of bleeding (≤ 1.5%) and laboratory evaluation of hemostasis including platelet count is not recommended in patients with liver cirrhosis since peri- or postprocedural bleeding cannot be predicted by alterations of these parameters [14]. However, bleeding complication rates still vary widely in the current literature and few representative studies have evaluated the association between bleeding and platelet count [15,16]. Furthermore, procedure-related death is almost always observed as a result of major bleeding.

Besides hemostasis parameters, several further factors are supposed to influence complication rates, including location of biopsy, the size and type of biopsy needle or the number of samples taken. However, the current data situation still remains unclear regarding the impact of these variables.

In this large retrospective analysis, a total of 12,117 percutaneous liver biopsies were analysed from 194 participating centres in Germany between 2018 and 2021.The aim of the following study was to identify and characterize the incidence of complications of liver biopsy, to identify potential risk factors for the occurrence of major complications, and to analyze the relationship between hemostasis parameters and peri-/postprocedural bleeding.

## Methods

### Data collection

This retrospective analysis was performed using data from the German Society of Interventional Radiology (DeGIR). Liver biopsies performed between January 1, 2018, and December 31, 2021, at 194 centers in Germany were included and retrospectively analyzed. Detailed information on each procedure was provided by the participating interventional centers in a web-based format and submitted to the DeGIR database using both symmetric and asymmetric coding techniques to ensure anonymization of the records. DeGIR database is a voluntary quality assurance registry, maintained by the German Society for Interventional Radiology. Participation in the registry is voluntary. During the study period, 194 centres reported all biopsies performed within the specified timeframe (2018–2021). Due to anonymization, the authors had no access to information that could identify individual participants. The study protocol and data analysis were approved by the local ethics committee of the Technical University of Munich (23-387S).

Information collected from the database included patient gender, age, needle size, lesion size, liver segment, number of samples collected, aPTT level, Quick level, and platelet count.

Post-liver biopsy complications were divided into minor and major complications within the first 24 hours after the procedure. Major complications included post-biopsy bleeding, visceral injury, and death. Minor complications included significant pain and peri-procedural hypotension.

### Hematological parameters

Normal range of aPTT was defined as 20–38 seconds. The normal range for International Normalized Ratio (INR) was defined as 0.8 to 1.1. In addition, a pathologic platelet count was defined as < 150,000 per microliter.

For the various epidemiological, clinical and hematological parameters, data were not always available from all patients. Thus, medians, ranges, and percentages were always calculated based on the corresponding available data, which were at baseline as follows: gender, n = 12117; age, n = 12115; aPTT, n = 11126; Quick-INR, n = 11332; platelet count, n = 11206; setting, n = 12117; liver segment, n = 8273; lesion size, n = 7426; sedation, n = 12117; local anesthesia, n = 12117; intubation anesthesia, n = 12117; imaging modality, n = 12117; needle size, n = 12117; number of biopsies performed, n = 2033.

### Statistical analysis

Categorical variables were reported as frequencies (percentages) and continuous variables as median (range). Variables with non-normal distribution were analyzed with the Mann-Whitney *U* test and expressed as median and interquartile range. Categorical variables were compared using the chi-square or Fisher exact test, as appropriate, and expressed as frequencies and percentages. A p-value of ≤ 0.05 was considered statistically significant. Variables showing p < 0.05 in the univariate model were analyzed in a multivariate logistic regression model. Odds ratios (ORs) and 95% CIs (Confidence Intervals) were calculated for the independent predictive factors of postprocedural complications. Statistical analysis was performed using IBM SPSS 26.0 statistical software package (SPSS/IBM, Munich, Germany).

## Results

### Patient demographics

A total of 12117 liver biopsies were performed during the study period. The median age of the study cohort was 70 years (range, 10–107 years) and 50% were male (n = 6064). All liver biopsies were performed percutaneously. Ultrasound guidance was used in 37.2% (n = 4511) of the biopsies, while 62.8% were performed CT-guided (n = 7606). Needle calibers ranged from 8 to 22G, with 18G (n = 7360; 60.7%) being the most commonly used. A total of 11452 procedures (94.5%) were performed in the inpatient setting and only a minority (5.5%) were performed in the outpatient setting. In most cases (n = 11704; 96.6%), local anesthesia of the subcutaneous tissue and the liver capsule was used. Conscious sedation was used in n = 252 (2.1%) of patients and n = 312 (2.6%) of cases were performed under intubation anesthesia.

Pathological values for aPTT, Quick-INR and platelet count were observed in 9.0% (n = 997 of 11126), 10.3% (n = 1171 of 11332) and 13.5% (n = 1523 of 11206), respectively.

The number of samples taken ranged between n = 1 and n = 15 with n = 3 being the most frequently taken number of samples (55.8%). In most cases, targeted biopsy of solitary hepatic lesions was performed (98%). Non-targeted biopsy for evaluating severity or genesis of liver disease was conducted in 235 patients (1.94%).

Out of 12117 liver biopsies, adverse events were observed in n = 235 patients, resulting in a complication rate of 1.9%.

#### Liver biopsy complications.

Minor complications were observed in 40 patients (0.33%) and major complications in 195 patients (1.61%). With n = 166 events observed, post-procedural bleeding was the most common major complication among all liver biopsies. Other major complications were pneumothorax (n = 26; 0.21%) and inadvertent organ injury (n = 3; 0.02%). A total of 3 deaths were recorded in the entire cohort, all due to massive post-procedural intraperitoneal bleeding. Total number of complications in patients undergoing liver biopsy in our cohort are shown in Table 1.

**Table 1:** Total number of complications in patients undergoing liver biopsy (n = 235).

	n (%)
Minor complication, n (%)	40 (0.33)
Vasovagal or procedural hypotension, n (%)	7 (0.06)
Others (including pain and medication side effect), n (%)	33 (0.3)
Major complication, n (%)	195 (1.6)
Bleeding, n (%)	166 (1.4)
Venous Bleeding, n (%)	54 (0.4)
Arterial Bleeding, n (%)	74 (0.6)
Parenchymal Bleeding, n (%)	38 (0.3)
(Hollow) Organ injury, n (%)	3 (0.02)
Pneumothorax, n (%)	26 (0.2)
Death (all bleeding), n (%)	3 (0.02)

### Analysis of pre-biopsy clinical and hematological differences among all major complications

Subjects with major complications were marginally older with a median age of 72 years compared to 70 years in all other subjects (p = 0.22). Regarding the gender as further baseline characteristic, no significant difference could be detected (p = 0.44).

Regarding pre-biopsy coagulation parameters, no significant differences could be observed between those with major complications and those without: pathological aPTT and INR values were observed in 10.3% and 13.3% of patients with major complications compared to 8.2% and 9.6% in those without (p = 0.37 and 0.10, respectively). In contrast, decreased platelet count was significantly more frequently observed in patients who experienced major complications compared to those without (20.0% vs. 12.4%, respectively; p = 0.002).

Analyzing the occurrence of major complications by different modalities, subjects who underwent CT-guided biopsies, significantly more frequently experienced major complications compared to those who were punctured ultrasound-guided (1.31% vs. 0.30%, respectively; p < 0.001). However, patients undergoing CT-guided biopsies were significantly less likely to have pathological platelet counts than those who underwent ultrasound-guided biopsies (11.3% vs 13.6%, p < 0.001).

Moreover, an insignificant trend towards a higher rate of major complications was observed in punctures conducted by > 3 liver biopsies compared to those performed by 1–3 biopsies (1.7% vs. 2.0%, p = 0.35). In contrast, the needle size did not impact the rate of complications: biopsies performed by a larger needle size (>16G) resulted in a marginally lower rate of relevant complications (1.6%) compared to those conducted by a smaller needle size (10-16G; 1.7%, p = 0.71).

Assessing the frequency of major complications when biopsying different liver segments, significant differences could be observed (p < 0.001): highest rates of major complications were detected, when liver segment VII and VIII were biopsied (0.8% and 0.6%). In contrast, punctions performed in liver segments I and II were comparatively low (0% and 0.1%). The size of punctured lesions did not impact the general complication rate (p = 0.34). Demographic and clinical factors associated with major complications are shown in Table 2.

**Table 2:** Demographic and clinical factors associated with major complications (n = 195).

	Major complication (n = 195)	No major complication (n = 11922)
Males, n (%)	96 (47)	5927 (50)
Age (years), median (range)	72 (26-84)	70 (10-107)
Pre-biopsy laboratory data		
Abnormal aPTT, n (%)	20 (10)	979 (8)
Abnormal INR, n (%)	26 (13)	1145 (10)
Abnormal platelet count, n (%)	39 (20)	1484 (12)
Biopsy characteristics		
Needle size	18 (12–20)	18 (10–24)
Modality (CT/Ultrasound)	159 (82)/ 36 (18)	7252 (61)/ 4475 (38)
Outpatient setting	6 (3)	659 (6)
Number of biopsies taken	3 (1–10)	3 (1–15)
Lesion characteristics		
Liver segment (n = 8237)		
Liver segment I, n (%)	0 (0)	90 (1)
Liver segment II, n (%)	16 (8)	786 (7)
Liver segment III, n (%)	10 (5)	819 (7)
Liver segment IV, n (%)	12 (6)	1287 (11)
Liver segment V, n (%)	20 (10)	956 (8)
Liver segment VI, n (%)	22 (11)	1281 (11)
Liver segment VII, n (%)	66 (34)	1302 (11)
Liver segment VIII, n (%)	49 (25)	1521 (13)
Lesion size (mm), median (range)	22 (9-691)	21 (5-736)

Finally, we conducted logistic regression analysis in order to identify independent predictors of major complications. Based on a univariate analysis, pathological platelet count, the imaging modality used as well as the liver segment biopsied were significantly associated with the occurrence of major complications. In a following multivariate analysis, all of these three variables (platelet count, imaging modality and liver segment) could be identified as predictive factors of major complications (Table 3).

**Table 3:** Univariate and multivariate analysis of factors associated with major complications (n = 195).

	All major Complications (n = 195)	Univariate *p* value	Multivariate OR (95% CI)	*p* value
Males (%)	96 (47)	0.44		
Age (years), median (range)	72 (26-83)	0.22		
Pre-biopsy laboratory data				
Abnormal aPTT, n (%)	20 (10)	0.37		
Abnormal INR, n (%)	26 (13)	0.10		
Abnormal thrombocytes, n (%)	39 (20)	0.002	2.11 (1.35 - 3.30)	<0.001
Biopsy characteristics				
Puncture needle size,	18 (12–20)	0.70		
median (range)				
Imaging modality (CT/Ultrasound), n (%)	159 (82)/36 (18)	<0.001	5.02 (4.12-6.18)	<0.001
Setting, n (%)	6 (3)	0.14		
Number of biopsies taken, median (range)	3 (1–10)	0.91		
Lesion characteristics				
Liver segment location		<0.001	4.12 (3.15–5.29)	0.002
Lesion size, n (%)	22 (6-691)	0.34		

### Analysis of pre-biopsy clinical and hematological differences among bleeding complications

Comparing the group of bleeding complications with those without bleeding, several differences were identified. Platelet count was significantly more frequently pathological in the bleeding group (p < 0.001). Moreover, as already observed when analyzing the differences in subjects with general complications, the imaging modality was observed to impact the occurrence of bleeding complications (p < 0.001). Finally, bleeding complications significantly differed by the liver segments biopsied (p < 0.001). All remaining parameters were not significantly different in subjects with and without bleeding complications. Demographic and clinical factors associated with bleeding complications are presented in Table 4.

**Table 4:** Demographic and clinical factors associated with bleeding complications (n = 166).

	Bleeding complication (n = 166)	No bleeding complication (n = 11951)
Males, n (%)	82 (49)	5941 (50)
Age (years), median (range)	72 (30-84)	70 (10-107)
Pre-biopsy laboratory data		
Abnormal aPTT	18 (11)	981 (8)
Abnormal INR, n (%)	22 (13)	1149 (10)
Abnormal platelet count, n (%)	38 (23)	1485 (12)
Biopsy characteristics		
Needle size	18 (12–20)	18 (10–24)
Modality (CT/Ultrasound)	138 (83)/ 28 (17)	7273 (61)/ 4485 (38)
Outpatient setting	6 (4)	659 (6)
Number of biopsies taken	3 (1–9)	3 (1–15)
Lesion characteristics		
Liver segment		
Liver segment I, n (%)	0 (0)	90 (1)
Liver segment II, n (%)	14 (8)	788 (7)
Liver segment III, n (%)	10 (6)	819 (7)
Liver segment IV, n (%)	11 (7)	1288 (11)
Liver segment V, n (%)	15 (9)	961 (8)
Liver segment VI, n (%)	17 (10)	1286 (11)
Liver segment VII, n (%)	56 (34)	1312 (11)
Liver segment VIII, n (%)	43 (26)	1527 (13)
Lesion size, median (range)	22 (9-598)	21 (5-736)

A consecutive multivariate stepwise logistic regression analysis revealed that a platelet count < 150,000 per microliter, liver segment location of punctured lesions and imaging modality were identified as independent predictors of bleeding risk (Table 5).

**Table 5:** Univariate and multivariate analysis of factors associated with bleeding complications (n = 166).

	All bleeding Complications (n = 166)	Univariate *p* value	Multivariate OR (95% CI)	*p* value
Males (%)	82 (49)	0.94		
Age (years), median (range)	70 (30-84)	0.51		
Pre-biopsy laboratory data				
Abnormal aPTT, n (%)	18 (11)	0.22		
Abnormal INR, n (%)	22 (13)	0.12		
Abnormal thrombocytes, n (%)	38 (23)	<0.001	2.98 (2.20-3.71)	<0.001
Biopsy characteristics				
Puncture needle size, median (range)	18 (12–20)	0.66		
Imaging modality (CT/Ultrasound), n (%)	138 (83)/28 (17)	<0.001	5.24 (4.31-6.42)	<0.001
Setting, n (%)	6 (4)	0.29		
Number of biopsies taken, median (range)	3 (1–9)	0.81		
Lesion characteristics				
Liver segment location		<0.001	4.33 (3.57-5.24)	0.007
Lesion size, n (%)	22 (9-598)	0.31		

## Discussion

The comprehensive analysis of liver biopsies performed in this study revealed important information about the role of coagulation parameters in predicting general and bleeding-specific complications. Among these parameters, platelet count is a particularly important factor, bolstering previous research [17].

Our findings contraststudies showing no increased risk of bleeding complications with pathological platelet counts [18–20]. According to the recently published EASL clinical practice guidelines, both percutaneous and transjugular biopsy are classified as procedures associated with a low risk of bleeding (≤ 1.5%), and laboratory evaluation of hemostasis, including platelet count, is not recommended in patients with liver cirrhosis [14]. The discrepancy with our findings highlights the complexity of liver biopsy procedures and the need for individualized risk assessment strategies [19].

Interestingly, the impact of mild hemostatic abnormalities on bleeding risk has been the subject of debate. One study evaluating patients with mild elevations in aPTT suggested that prophylactic transfusions may not be necessary in patients with platelet counts greater than or equal to 50 x 10^9/L, as the frequency of bleeding complications was not significantly different from patients with normal parameters [21]. This finding supports our results that aPTT and Quick-INR did not show significant predictive value for bleeding complications.

In terms of pathologic platelet count and complication rate, our findings are consistent with previous studies that have highlighted the importance of platelet count as a key risk factor. A significant increase in bleeding complications and deaths with a platelet count of 70 × 10^9/L or less was already discussed in the past, highlighting the critical need for careful pre-procedure platelet count assessment [22]. Furthermore, Seeff et al. suggest a graded risk based on platelet count, with a marked increase in complications at counts ≤60,000/mm³ [23]. This reinforces our conclusion that monitoring and, if necessary, correcting the platelet count prior to biopsy is essential to minimize risk [24].

Regarding the role of needle size and coagulation parameters, our study contrasts previous studies [25,26]. Our analyses show that needle size and coagulation parameters do not significantly influence the rate of bleeding complications during liver biopsy.

CT-guided liver biopsies have become an important component in the diagnostic evaluation of liver disease, offering advantages over blind percutaneous biopsies in terms of imaging guidance and accuracy.

Compared with ultrasound-guided procedures, CT-guided biopsies were associated with a higher incidence of complications. CT guidance may be chosen more often for complicated cases or lesions that are difficult to access, which may explain the higher complication rate compared to ultrasound guidance. Interestingly, despite the lower proportion of patients with a pathological platelet count in the CT-guided group, the complication rate remained higher, possibly highlighting the complexity of cases typically managed under CT guidance. As noted before, this observation suggests that the lower proportion of pathological platelet counts in the CT-guided group may partly reflect a selection bias, as patients undergoing ultrasound-guided biopsies were possibly selected for less complex cases. However, a study of kidney biopsies performed under ultrasound or CT guidance found that complication rates were significantly higher for CT-guided biopsies compared with ultrasound-guided biopsies [27]. Although this refers to kidney biopsies, similar trends may also apply to liver biopsies.

In addition, the liver segment biopsied played a critical role in determining complication rates. Biopsies performed in segments VII and VIII had higher rates of major complications, likely due to the anatomical and functional characteristics of these regions. These segments are subject to significant respiratory motion, which can lead to parenchymal damage from needle shear forces.

In particular, older patient age did not correlate with a higher complication rate. This conclusion is in line with the existing literature. Previous studies suggest that age is not a predictive risk factor for complications following elective surgery in elderly patients, with other factors such as frailty, cognitive impairment and smoking being more important [28]. Therefore, further research highlights the complexity of age as a risk factor and emphasizes the importance of considering the type of procedure and whether it is performed in an emergency setting, as well as the patient’s overall health and physiological status [29,30]. In summary, although age could be a factor influencing the likelihood of complications after image-guided procedures, it is not the only determinant and its impact is relatively small compared to other factors. This highlights the need for a comprehensive approach to patient risk assessment that takes into account a number of factors.

A major limitation of this study is the categorical nature of the data collected in the DeGIR registry. For instance, platelet counts were categorised as normal (≥150,000/ µ L) or pathological (<150,000/ µ L), without further stratification into thresholds such as < 60,000/ µ L. Additionally, the dataset does not include information on whether corrections for coagulopathy were made before biopsy. These limitations should be addressed in future prospective studies with more detailed data collection. Moreover, the registery is based on voluntary documentation of interventions and might therefore be prone to register bias. Yet, it is of note that there is no data set published with similar intervention numbers.

In conclusion, our study highlights the importance of a nuanced approach to patient evaluation for liver biopsy procedures. While platelet count appears to be a significant predictor, the utility of aPTT and Quick-INR values may be more limited or require additional factors for effective risk stratification. These findings underscore the need for careful patient evaluation and procedural planning, emphasizing thorough preprocedural assessment and interprofessional coordination to optimize patient safety and outcomes. Although the study is comprehensive in nature, prospective studies may provide further insight into the multifaceted nature of liver biopsy complications. Moreover, the results may help refine biopsy protocols, improve patient safety, and guide future research in this area.

**Key Points**

- Study investigates complication rates in percutaneous liver biopsies
- Platelet count, imaging modality and the liver segment biopsied significantly influence the complication rate
- Needle size and coagulation parameters do not significantly affect complication rates
